# Supplementary material for: Advanced biofilm analysis in streams receiving organic deicer runoff
Source: PLoS One. 2020 Jan 22;15(1):e0227567. doi: 10.1371/journal.pone.0227567 (PMC6975536; doi:10.1371/journal.pone.0227567)
Supplement: S1 Appendix — (DOC) [file pone.0227567.s001.doc]

# **S1. Appendix. Additional details on materials and methods.**

## Water data

### **Dye tracer studies**

From 2005 through 2008, dye tracers were used to determine time of travel between DS1-gage and DS3-gage over a variety of streamflow conditions. A pump was used to inject Rhodamine WT dye into the stream just below the gage-control weir at DS1-gage, and a fluorometer was used at DS3-gage to detect the dye. The time of travel between injection at DS1-gage and the peak of the plume at DS3-gage was recorded along with streamflow at DS3-gage, and a relation describing the flow dependence of travel times between the two gages was established (see the Water Data methods section in the main text).

### **Quality control: water-quality samples**

Quality control samples were collected in conjunction with both the grab and the flow-weighted composite samples. For the grab samples, eight field replicates were analyzed for all nutrients and chemical oxygen demand (COD). Relative percent differences ranged from 0.4 to 15% for nitrate plus nitrite, 0 to 63% for total Kjeldahl nitrogen (TKN), 0 to 8% for total phosphorus, and 0 to 10% for COD (with the exception of one COD sample pair above and below the reporting limit at 10 and less than 8 mg/L, respectively). For the flow-weighted composite samples, four field blanks (two per year) were analyzed for COD, two were analyzed for PG, and one was analyzed for acetate; analytical results were less than the reporting levels (8, 20, and 5 mg/L, respectively) for all constituents in all samples.

## Biofilm field surveys

Biofilm field surveys were performed during 2009-2011 at each of the four main sites using methods adapted from established rapid periphyton survey and stream habitat protocols [1–3]. At each site, short reaches spanning twice the mean wetted channel width were established, and five cross-sectional transects were spaced at equidistant intervals along each reach. Data were collected from within the wetted channel at equidistant points along transects and totaled approximately 50 points per reach. Point-level data collection included assessments of visual biofilm structure, biofilm coloring, and biofilm thickness; reach-level data collection included assessments of the average percent cover and thickness of ice, as well as average wetted width and reach length. Biofilms were categorized into one of four operational classes according to dominant color and morphology: ‘soft algae’, ‘transition’ (soft algae-heterotroph mix), ‘heterotrophs’, and ‘diatoms’ (Fig A). Diatom categorizations were further assisted by photographs taken during each trip, and microscopy descriptions for examined samples. Biofilm thickness was measured using a meter stick, and observable biofilms with thicknesses smaller than 0.1 cm were assigned a value of 0.05 cm; biofilms less than 0.05 cm in thickness were considered below the limit of detection and classification and were reported as <0.05 cm. In cases where biofilms were filamentous in morphology, thickness measurements reflect the vertical distance from the streambed substrate to the top of the biofilm assemblage, and not the length of biofilm filaments themselves. Apart from a high flow event that interrupted the February 2011 trip, data were collected from all four sites during every trip.

**
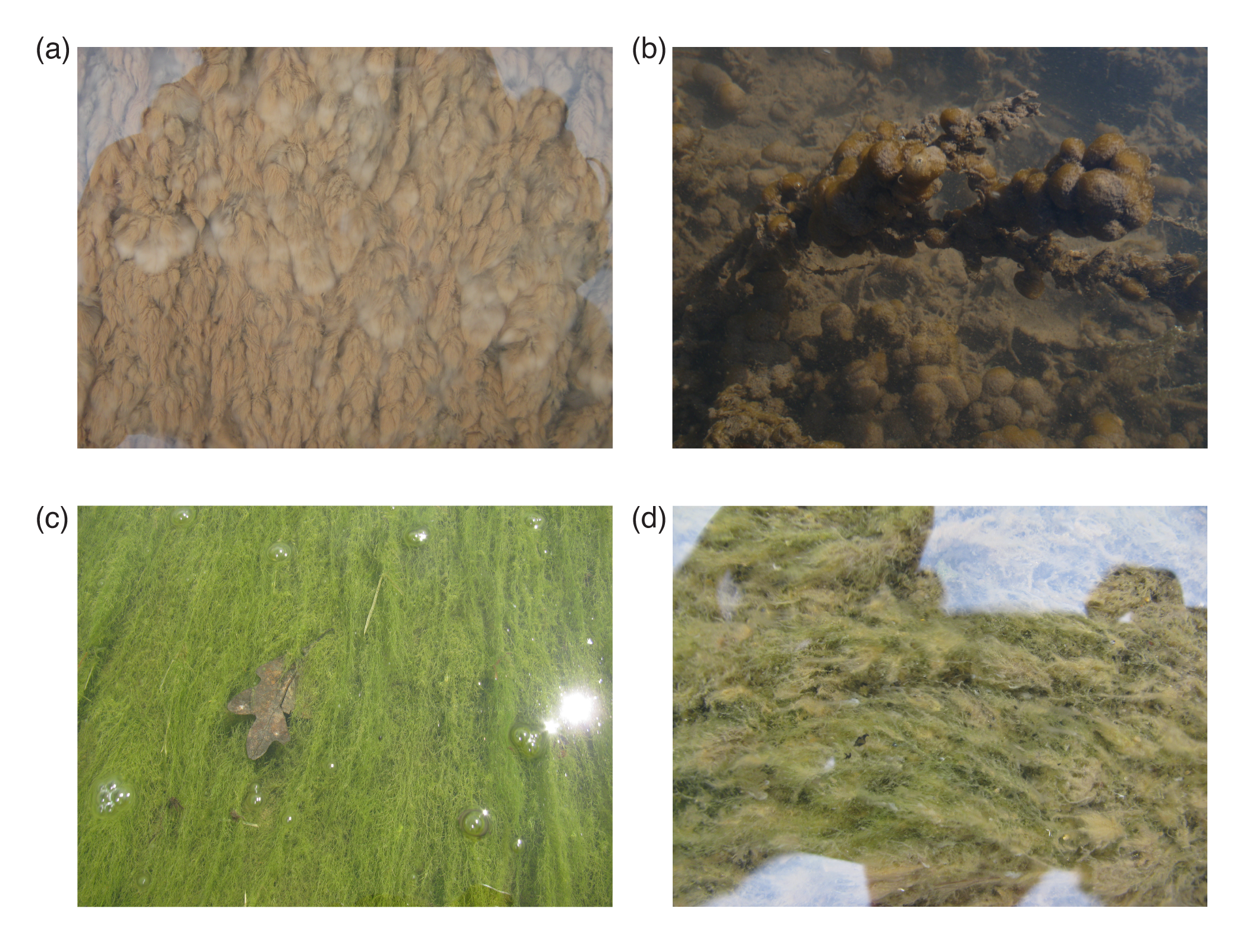
**

**Fig A.** **Photographs of biofilm classes encountered at sites.** (a) heterotroph-dominated biofilm (DS1 reach on December 23, 2009); (b) diatom-dominated biofilm (covering macrophytes; US1 reach on March 29, 2011); (c) soft-algae-dominated biofilm (DS3 reach on June 17, 2010); and (d) transitional biofilm (i.e., mix of soft algae and heterotrophs; DS1 reach on June 18, 2010).

Reaches at each site were located in runs in exposed (i.e., not heavily shaded) areas of the channel, in an effort to minimize flow/turbulence and sunlight exposure differences. The physical characteristics of the channels did, however, vary somewhat between sites (Fig B). US1 and DS2 sites had soft-sediment streambeds (silt and sand, respectively), with harder substrates along the bank (gabion and rip rap, respectively) and relatively low streamflow velocities. DS1 and DS3 were concrete-lined channels with moderate streamflow velocities. Transect locations at all four sites remained as static as possible throughout the biofilm survey period (i.e., 2009-2011).

## Biofilm sample collection and laboratory analyses

### **Sample collection**


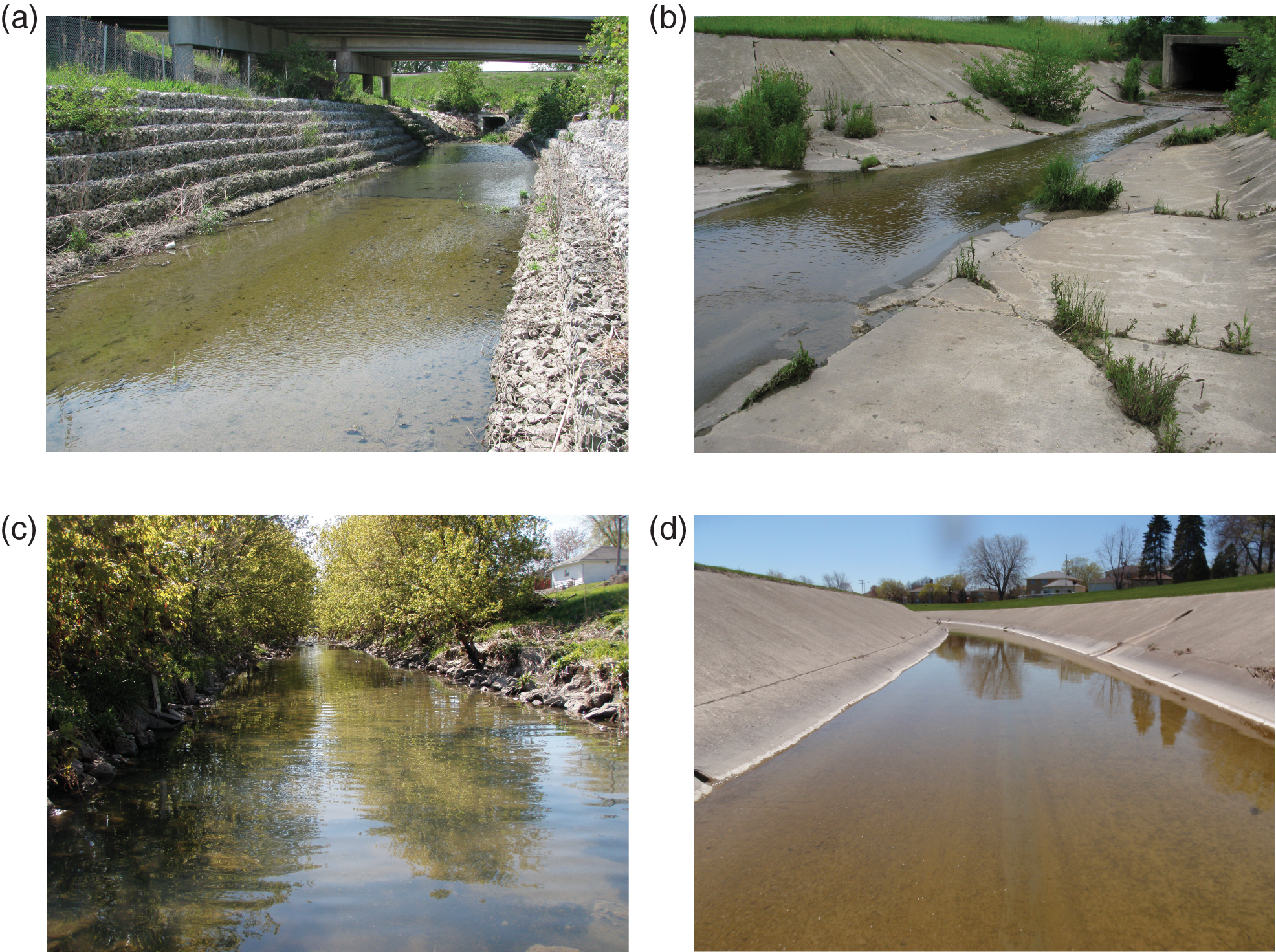


**Fig B.** **Photographs of biofilm survey reaches at each of the four main sites.** (a) US1, looking downstream; (b) DS1, looking upstream; (c) DS2, looking upstream; and (d) DS3, looking upstream.

### **DNA extractions**

Genomic DNA was extracted (from approximately 200 mg of wet sample) using three methods to ascertain the best protocol for optimal DNA recovery. These methods included (1) hot phenol chloroform, (2) a cetyl trimethyl ammonium bromide method from Omega Bio-tek (D3373-01; Norcross, Georgia, USA), and (3) Qiagen DNA QIAamp system (Hilden, Germany). Multiple methods were necessary as these types of biofilm samples are not typical due to excessive extracellular polysaccharides that may exist within the matrix. In all cases the samples were subject to two enzymatic digestions including a mix of mutanolysin, lysozyme, achromopeptidase, and lysostaphin (currently sold as MetaPolyzyme; Millipore Sigma, St. Louis, Missouri, USA) followed by a proteinase K digestion. After enzymatic digestion, extraction buffer (depending on the method) was combined with an AlO_3_ abrasive and homogenized with an MP Biomedicals FastPrep-24 (Santa Ana, California, USA) for 60 seconds. After the homogenization step all DNA extraction methods were completed following manufacturer-recommended protocols and eluted in 30 µL of 10mM Tris. The resulting DNA was quantified using the NanoDrop ND-1000 spectrophotometer (ThermoFisher Scientific; Waltham, Massachusetts, USA). Of the three DNA methods tested, the Omega Bio-tek method showed the highest DNA yields and was used exclusively for samples collected the second year. This kit utilized the same fundamental extraction approach shown to be effective for environmental samples elsewhere [4].

### **Microarray**

From each of 11 samples, approximately 50 ng of DNA were polymerase chain reaction (PCR) amplified using universal 16S rRNA gene primers (27F and 1492R; Table A). Samples were cleaned and concentrated using Pall Nanosep® 30K spin filters (Port Washington, New York, USA). Approximately 640 ng of sample were then fragmented and labeled using DNase I (0.025 U/µL) and TdT (30 U/µL; Affymetrix GeneChip WT Terminal Labeling Kit: 900670). Efficiency of the biotin labeling reaction was verified using NeutrAvidin (10 mg/mL) with a gel-shift assay. Samples were injected into Affymetrix G2 PhyloChip phylogenetic arrays and placed in the Affymetrix Genechip® Hybridization Oven 640 at 48°C for 16-18 hours overnight. A full description of the G2 PhyloChip design is provided elsewhere [5–7]. Hybridized arrays were washed and stained with streptavidin-phycoerythrin followed by sequential incubations with biotin-couple polyclonal anti-streptavidin antibody and streptavidin-phycoerythrin as a fluorescent amplification step. Finally, arrays were scanned with the 7G Affymetrix Genechip® Scanner 3000.

**Table A. Targeted regions and associated primers used for quantitative real-time polymerase chain reaction (qPCR) and sequencing efforts.**

| Target region | Primer name | Primer sequence | Reference |
| --- | --- | --- | --- |
| 16S rRNA gene | 27F | AGAGTTTGATYMTGGCTCAG | Nercessian et al., 2005 [8] |
|  | 519F | GTGCCAGCMGCCGCGGTAA | Modified from Burggraf et al., 1997 [9] |
|  | 536R | GTATTACCGCGGCKGCTG | Ruff-Roberts et al., 1994 [10] |
|  | 1055F | ATGGCYGTCGTCAGCTCGT | Designed for current study |
|  | 1330F | CTAGCGATTCCGACTTCA | Designed for current study |
|  | 1390R | ACGGGCGGTGTGTRCAA | Mao et al., 2012 [11] |
|  | 1492R | GGYTACCTTGTTACGACTT | Lane, 1991 [12] |
| 23S | 37R | GCCAAGGCATCCRCC | Designed for current study |
|  | 127R | GGTTBCCCCATTCRGAH | Designed for current study |
| *sthA* gene^a^ | 134F | GCCCTCGTCACKCCCATGCTG | Designed for current study |
|  | 730F | AAGTGGGGCGTGAAGCCG | Designed for current study |
|  | 1041R | ACCACGTTCGGATAGCCCTC | Designed for current study |

^a^Final *sthA* DNA consensus sequence determined by comparison of whole metagenome sequencing data to the Suzuki *sthA* GenBank sequence (AB050640.1) is shown below; green highlighting indicates qPCR forward primer sites while red highlighting indicates reverse qPCR primer sites.

TCAGATGCGCATAGCCCTCGTCACTCCCATGCTGCCCGTTCCGCACGACCAGACGCGGGGCCGCTACATCCACGAAACCGCCCGCTCGCTGGGCAAGCTCGCCACGGTGAAGACCTTCTTCCAGACGATGCAGTACCCGAAGGTGCCCGGGGCCTGCGCGCCGCGCAGCTTCATCTACGGCTCGGTCGGCCCGGACTACACGCTGGACGGCTGCGATGTCGAGGCCTTCACCTTCATGGGGCGTGCCGGTGGTCAGCCGCGGCTACAACGGCCACATCGCCAGCCTCGCGCTGACGCCGCGCGTGCGCCGCTTCAAGCCGGACCTGGTGCTGGCCTACTGGGTCTATCCGGACGGGTTTGCCGCGATGCGCACGGCGCGGGCGCTGGGCGTGCCGTGCATCGTCGGCGCGCTGGGCTCGGACATCCATGTCCGCTCGGGCGTGAACGAGAAGATGACACGCCGCACCATCGGCGGCATCGACGCGCTGCTGACGGTGAGCGAGGCGATGCGCCAGTACGCGATCCGCGAGTTCGGCGCGCCAGCCGAGCGGGTCCACACCATCATCAACGGCTTCAACACCGCCGTCTTCAAGCCGCTCGACCAGCCGTCGCTGCGCGCGAAGTGGGGCGTGAAGCCGGACGAGAAGATGATCGTCTACGTCGGCCGCTTCGTCGAAGCCAAGGGCATGCGCGAGCTGATCACCGCCTTCCAGCAACTCGCCAAGGACGACCCCAAGGTGACGCTGGCCCTCGTCGGCGACGGCGTGATGAAGGCGGAGCTGATGGCGCTGGTGGCGTCGACCGGGCTGACCGAGCGCGTCCATCTGCCGGGTGGTCAGGCGCCCGAGCAGGTCGCCGAGTGGATCAACGCTGCCGACGTGCTGACGCTGCCGAGCTGGAGCGAGGGCTATCCGAACGTGGT

The PhyloChip G2 .CEL files were analyzed by Second Genome (South San Francisco, California, USA) using an empirical approach to define unique operational taxonomic units (eOTUs). The criteria for scoring the probe-level fluorescence intensity (FI) and the process by which individual probes are clustered into probesets, aka eOTUs, are described in detail elsewhere [13]. Briefly, in the eOTU analysis taxonomically related probes were clustered into probesets when pairwise correlations between log2 transformed FI values were ≥0.85. Probesets were annotated with the 2012 Greengenes taxonomy using Naive Bayesian scoring and a ≥80% bootstrapped confidence threshold [14,15]. The mean log2 FI among the multiple probes for each eOTU, referred to as the hybridization score (HybScore), was calculated for each sample. A binary score for each eOTU was assigned a 1 if 80% or more of the probes in that set were responsive in a given sample (present), otherwise a 0 was assigned (absent).

The eOTU abundances from the analysis of PhyloChip data were further analyzed using MeV (MultiExperiment Viewer) in the TM4 software [16]. Hierarchical clustering of microbial genera utilized the average linkage method and Pearson Correlation distance metric [17,18]. Additionally, a principal coordinates analysis (PCoA) was performed to assess relations between samples using Fast UniFrac [19]. Specifically, PCoA plots were constructed based on a weighted UniFrac dissimilarity matrix [20], which accounts for both phylogenetic distance between eOTUs and eOTU abundances.

### **Whole metagenome sequencing**

Whole genome metagenomic sequencing libraries were synthesized from 500 ng of genomic DNA by first fragmenting with an Illumina TruSeq nebulizer (San Diego, California, USA) and using 100 ng for labelling with an Illumina TruSeq V1 DNA kit. Final libraries were checked for size distribution, concentration, and quality using the Agilent 2100 Bioanalyzer (Santa Clara, California, USA), Qubit spectrofluorometer (Thermo Fisher Scientific; Waltham, Massachusetts, USA), and qPCR using KAPA Biosystems quantification reagents (Roche; Basel, Switzerland). Sequencing was performed using an Illumina HiSeq 1500 equipped with a paired end 2x100bp flow cells to a depth of 200 million reads per sample.

Three primers were developed by aligning the whole metagenome sequencing (WMS) data to the Suzuki *sthA* GenBank sequence (AB050640.1; [21]) and building a consensus sequence (Table A) using DNASTAR SeqMan NGen 12.3.1 (Madison, Wisconsin, USA). This consensus sequence was used to develop primers using NCBI Primer-BLAST software (Bethesda, Maryland, USA) and DNASTAR SeqBuilder Pro. Due to the high GC content of the target gene, primer Tms were between 61 and 64 degrees Celsius. Primers were validated using qPCR and standard PCR assays, and the resulting 905 and 302 base pair amplicon products were validated using Sanger sequencing with the same forward and reverse primers. These developed primers were used for PCR, qPCR, and Sanger sequencing throughout this study to target the *sthA* gene sequence.

Raw sequences were converted to FASTQ format and uploaded to the One Codex (San Francisco, California, USA) metagenomics platform for taxonomic profiling using the NCBI RefSeq Complete Genomes, One Codex, and targeted loci databases [22]. The One Codex platform invokes classification using individual *k*-mers to the lowest common ancestor, and further selects the most consistent specific match across a given read. Results were filtered to genera having at least 3% classified reads.

### **Quantitative real-time PCR**

qPCR was performed using an Applied Biosystems 7900HT (Foster City, California, USA) with SYBR green chemistry to detect, quantify, and Sanger sequence the 16S-rDNA and *sthA* genes. Primers for the 16S-rDNA gene were previously described (Table A; [9,11]), and primers for the *sthA* gene were designed from the consensus sequence. Amplification and quantification of 16S rRNA gene and *sthA* DNA was performed using the universal primers 519F and 1390R (Table A) to generate an 800 bp amplicon and *sthA* primers 730F and 1041R (Table A) to generate a 302 bp amplicon, respectively. DNA input amounts for 16S rRNA gene were 0.1 ng/sample with thermocycling conditions of 98/50/72°C for 40 cycles. DNA input for *sthA* qPCR was increased because of potential for low copy number to 20 ng/sample with thermocycling conditions of 98/62/72°C for 45 cycles. All annealing temperatures were previously determined using manual PCR in a gradient thermocycler for -5 to +5 of the calculated thermal melting temperature (Tm) of the primers. Standard curves were generated using DNA from the DS1 sample collected on March 18, 2010.

The *sthA* primers were designed around a very difficult region of the GC-rich *sthA* gene and had the unfortunate side effect of amplifying off-target amplicons. qPCR dissociation curves for the *sthA* analyses showed peaks at 80, 86, 88, and 90°C. Resulting amplicons were run on a gel, bands were extracted and sequenced via Sanger sequencing, and comparisons were run against NCBI GenBank NT to identify the amplicons. This showed that the targeted qPCR product matching *Sphaerotilus natans sthA* glycosyltransferase (GenBank ID: AB050640.1) were associated with the Tm peaks at 80°C and 90°C, while off-target amplicons were associated with Tm peaks at 86°C and 88°C. The multiple peaks observed for the on-target amplicon were presumably a result of non-linear melting of this GC-rich amplicon. Genomic copy numbers for *sthA* were obtained by assessing the fraction of the signal represented by products with Tms of 80°C and 90°C and multiplying this fraction by the total sample signal to correct for the portion resulting from target amplicons.

### **Isolating and sequencing pure strains of sheathed bacteria**

Culturing on the May 2014 sample was done on three media: (1) a modified Casitone Glycerol Yeast Autolysate (CYGA) medium (ATCC 1103; Manassas, Virginia, USA) with trace vitamins, (2) Reasoner’s 2A (R2A) agar medium supplemented with BG-11 media (Millipore Sigma; St. Louis, Missouri, USA), and (3) 0.02% Proteose Peptone #3 (Becton, Dickinson and Company, Franklin Lakes, New Jersey, USA) agar medium. Isolations were conducted by both the University of Vermont and Yokohama National University, Japan. Over the period of 30 days, cultures were examined daily under a dissecting microscope until filamentous bacteria were observed. Colonies were purified using a micromanipulator and sub-cultured until pure (Fig C). A total of 23 different colonies were isolated. In order to determine whether they were clones, DNA was extracted from all 23 cultures and two sets of standard 16S rRNA gene primers (519F/1390R and 27F/1492R; [11]; Table A) were used to PCR amplify and Sanger sequence the 16S rRNA gene. The resulting sequences were aligned and determined to be from identical strains.


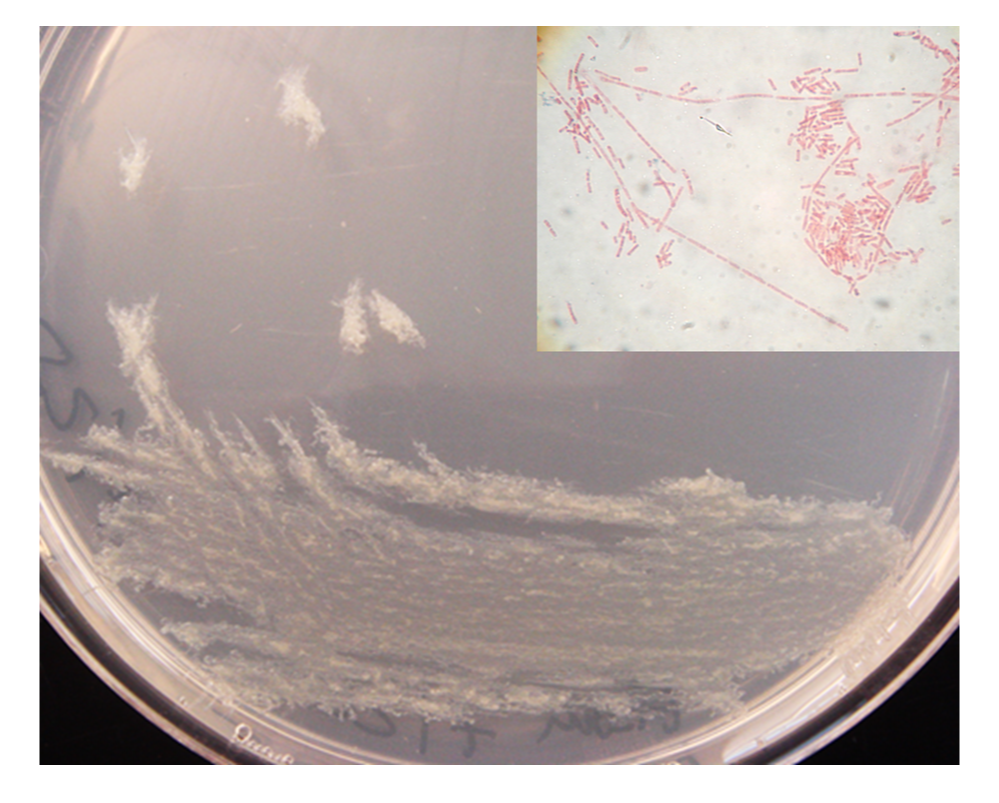


**Fig C.** **Pure culture of sheath biofilm bacterium *Sphaerotilus montanus* (strain KMKE) isolated during this study.** Isolate is shown here with Gram stain. Isolate has been deposited into the American Type Culture Collection as BAA-2725.

Subsequent Sanger DNA sequencing was used to fully characterize the entire 16S-ITS and partial 23S rRNA gene of the ribosomal operon [23]; DNA primers (including 16S primers: 27F, 519F, 536R, 1055F, 1330F, 1492R; and 23S primers: 37R, and 127R; Table A) provided genomic coverage from the 8 bp position of the 16S rRNA gene to 127 bp of a conserved region of 23S rRNA gene. The final sequences were used to assemble two contigs using ChromasPro (Technelysium Pty Ltd, Brisbane, Australia). Final sequence data were deposited into the NCBI NR database using the NCBI BankIt tool (accession numbers: KP096714.1 and KP096715.1) (Fig D). BLAST comparisons of the 16S-ITS sequence against the NCBI nucleotide NR database showed highest max score and identity (2599 and 100%, respectively) with *Sphaerotilus montanus* strain HS (GenBank NR_116396.1), and indicated this organism was likely a new strain of *Sphaerotilus montanus*. Additionally, the purified strain was deposited into the American Type Culture Collection (ATCC) as *Sphaerotilus montanus* strain KMKE (ATCC BAA-2725).

**
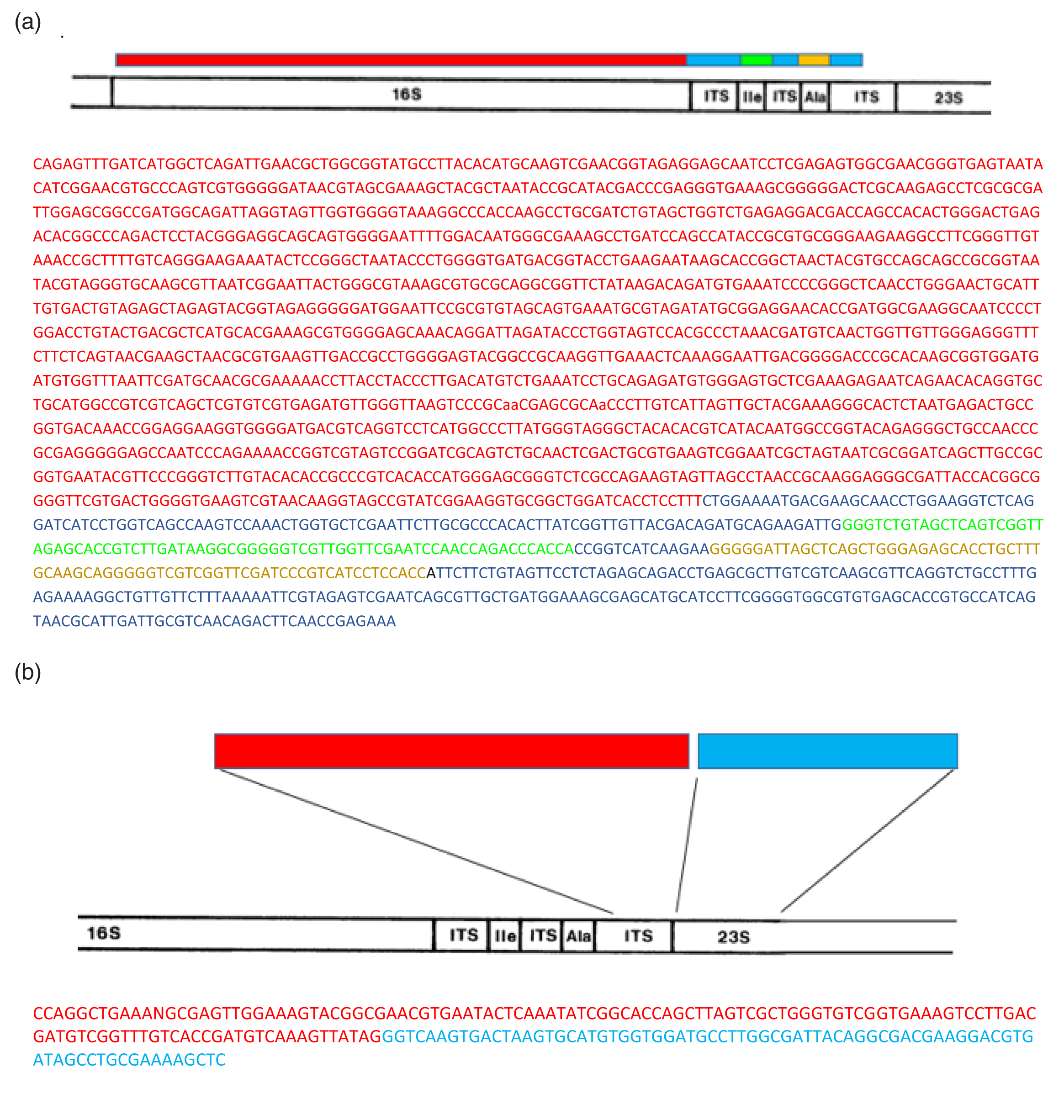
**

**Fig D.** **Sequences generated from the *Sphaerotilus montanus* isolate and deposited into NCBI NR database.** (a) nucleotide map and complete sequence of the 16S-ITS-IIe-Ala region, and (b) nucleotide map and partial sequence of the ITS-23S region.

Sanger sequencing was also performed on the *sthA* gene (using primers 134F and 1041R; Table A), and the resulting sequence was deposited into NCBI GenBank as a putative glycosyltransferase (*sthA*) gene (accession number: KF614510.1). It was also compared to the *sthA* sequence obtained from WMS of environmental samples.

### **Quality control: biofilm samples**

A replicate biofilm sample was collected from each of the four primary sites in association with biofilm sampling and comprised 9% of the total biofilm samples collected. Field replicate pairs were analyzed microscopically, as well as through qPCR techniques. Microscopy results for field replicate pairs were generally consistent with each other. The only difference occurred in the replicate pair collected from DS2 on June 1, 2011, where no sheath bacteria were observed in the regular sample, but they were observed in the replicate sample. Data associated with qPCR results showed greater variability among replicate pairs. Differences in qPCR 16S rDNA abundance for replicate pairs ranged from 3.9E-4 to 2.1E-3 ng (n=4). Differences in qPCR *sthA* DNA abundances for replicate pairs ranged from 1.1E-5 to 0.69 ng (n=3).

## Disclaimers

This publication does not necessarily represent the views of NIGMS or NIH but does represent the views of the U.S. Geological Survey. Any use of trade, firm, or product names is for descriptive purposes only and does not imply endorsement by the U.S. Government.

## References

1. Stevenson RJ, Bahls LL. Periphyton protocols. 2nd ed. Rapid Bioassessment Protocols for Use in Streams and Wadeable Rivers: Periphyton, Benthic Macroinvertebrates, and Fish, Second Edition. 2nd ed. Washington, D.C.: U.S. Environmental Protection Agency; Office of Water; 1999. pp. 6-1 through 6-22.

2. Stevenson RJ, Rollins SL. Chapter 34 - Ecological Assessments with Benthic Algae. Methods in Stream Ecology (Second Edition). San Diego: Academic Press; 2007. pp. 785–803. Available: http://www.sciencedirect.com/science/article/pii/B9780123329080500474

3. Fitzpatrick FA, Waite IR, D’Arconte PJ, Meador MR, Maupin MA, Gurtz ME. Revised Methods for Characterizing Stream Habitat in the National Water-Quality Assessment Program. 1998. Report No.: 98–4052. Available: http://pubs.usgs.gov/wri/wri984052/

4. Zhou J, Bruns MA, Tiedje JM. DNA recovery from soils of diverse composition. Appl Environ Microbiol. 1996;62: 316–322.

5. Brodie EL, DeSantis TZ, Joyner DC, Baek SM, Larsen JT, Andersen GL, et al. Application of a High-Density Oligonucleotide Microarray Approach To Study Bacterial Population Dynamics during Uranium Reduction and Reoxidation. Appl Environ Microbiol. 2006;72: 6288–6298. doi:10.1128/AEM.00246-06

6. Brodie EL, DeSantis TZ, Parker JPM, Zubietta IX, Piceno YM, Andersen GL. Urban aerosols harbor diverse and dynamic bacterial populations. Proc Natl Acad Sci. 2007;104: 299–304. doi:10.1073/pnas.0608255104

7. DeSantis TZ, Brodie EL, Moberg JP, Zubieta IX, Piceno YM, Andersen GL. High-Density Universal 16S rRNA Microarray Analysis Reveals Broader Diversity than Typical Clone Library When Sampling the Environment. Microb Ecol. 2007;53: 371–383. doi:10.1007/s00248-006-9134-9

8. Nercessian O, Fouquet Y, Pierre C, Prieur D, Jeanthon C. Diversity of Bacteria and Archaea associated with a carbonate-rich metalliferous sediment sample from the Rainbow vent field on the Mid-Atlantic Ridge. Environ Microbiol. 2005;7: 698–714. doi:10.1111/j.1462-2920.2005.00744.x

9. Burggraf S, Huber H, Stetter KO. Reclassification of the Crenarchaeal Orders and Families in Accordance with 16S rRNA Sequence Data. Int J Syst Evol Microbiol. 1997;47: 657–660. doi:10.1099/00207713-47-3-657

10. Ruff-Roberts AL, Kuenen JG, Ward DM. Distribution of cultivated and uncultivated cyanobacteria and *Chloroflexus*-like bacteria in hot spring microbial mats. Appl Environ Microbiol. 1994;60: 697–704.

11. Mao D-P, Zhou Q, Chen C-Y, Quan Z-X. Coverage evaluation of universal bacterial primers using the metagenomic datasets. BMC Microbiol. 2012;12: 66. doi:10.1186/1471-2180-12-66

12. Lane DJ. 16S/23S rRNA Sequencing. Nucleic acid techniques in bacterial systematics. New York: John Wiley and Sons; 1991. pp. 115–176.

13. Probst AJ, Lum PY, John B, Dubinsky EA, Piceno YM, Tom LM. Microarray of 16S rRNA gene probes for quantifying population differences across microbiome samples. Microarrays: Current Technology, Innovations and Applications. Norfolk, UK: Horizon Scientific Press and Caister Academic Press; 2014. pp. 99–119.

14. DeSantis TZ, Hugenholtz P, Larsen N, Rojas M, Brodie EL, Keller K, et al. Greengenes, a Chimera-Checked 16S rRNA Gene Database and Workbench Compatible with ARB. Appl Environ Microbiol. 2006;72: 5069–5072. doi:10.1128/AEM.03006-05

15. McDonald D, Price MN, Goodrich J, Nawrocki EP, DeSantis TZ, Probst A, et al. An improved Greengenes taxonomy with explicit ranks for ecological and evolutionary analyses of bacteria and archaea. ISME J. 2012;6: 610–618. doi:10.1038/ismej.2011.139

16. Howe E, Holton K, Nair S, Schlauch D, Sinha R, Quackenbush J. MeV: MultiExperiment Viewer. In: Ochs MF, Casagrande JT, Davuluri RV, editors. Biomedical Informatics for Cancer Research. Boston, MA: Springer US; 2010. pp. 267–277. doi:10.1007/978-1-4419-5714-6_15

17. Eisen MB, Spellman PT, Brown PO, Botstein D. Cluster analysis and display of genome-wide expression patterns. Proc Natl Acad Sci. 1998;95: 14863–14868.

18. Sokal RR, Michener CD. A statistical method for evaluating systematic relationships. Univ Kans Sci Bull. 1958;38: 1409–1438.

19. Hamady M, Lozupone C, Knight R. Fast UniFrac: facilitating high-throughput phylogenetic analyses of microbial communities including analysis of pyrosequencing and PhyloChip data. ISME J. 2010;4: 17–27. doi:10.1038/ismej.2009.97

20. Lozupone C, Hamady M, Knight R. UniFrac – An online tool for comparing microbial community diversity in a phylogenetic context. BMC Bioinformatics. 2006;7: 371. doi:10.1186/1471-2105-7-371

21. Suzuki T, Kanagawa T, Kamagata Y. Identification of a Gene Essential for Sheathed Structure Formation in *Sphaerotilus natans*, a Filamentous Sheathed Bacterium. Appl Environ Microbiol. 2002;68: 365–371.

22. Minot SS, Krumm N, Greenfield NB. One Codex: A Sensitive and Accurate Data Platform for Genomic Microbial Identification. bioRxiv. 2015; 23. doi:10.1101/027607

23. Boyer SL, Flechtner VR, Johansen JR. Is the 16S–23S rRNA Internal Transcribed Spacer Region a Good Tool for Use in Molecular Systematics and Population Genetics? A Case Study in Cyanobacteria. Mol Biol Evol. 2001;18: 1057–1069.
