# Supplementary material for: Advanced biofilm analysis in streams receiving organic deicer runoff
Source: PLoS One. 2020 Jan 22;15(1):e0227567. doi: 10.1371/journal.pone.0227567 (PMC6975536; doi:10.1371/journal.pone.0227567)
Supplement: S1 Table — Site name, identifiers, drainage area, distance upstream from the Wilson Park Creek at St. Luke’s Hospital gage (i.e., DS3-gage) for monitoring sites near Milwaukee Mitchell International Airport (MMIA) in Milwaukee, Wisconsin, USA. (DOC) [file pone.0227567.s002.doc]

**S1 Table.** **Site names and characteristics.** Site name, identifiers, drainage area, distance upstream from the Wilson Park Creek at St. Luke’s Hospital gage (i.e., DS3-gage) for monitoring sites near Milwaukee Mitchell International Airport (MMIA) in Milwaukee, Wisconsin, USA.

| **Site name** | **NWIS^a^ station identification code** | **Site identifier** | **Drainage area**  **(km^2^)** | **Distance upstream from DS3-gage site**  **(km)** |
| --- | --- | --- | --- | --- |
|
| Edgerton Channel near Pennsylvania Avenue | 040871474 | US1 | 2.1 | 8.66 |
| Wilson Park Creek at MMIA outfall 7 gage | 040871475 | DS1-gage | 7.7 | 6.06 |
| Wilson Park Creek at MMIA downstream from outfall 7 gage | (040871475) | DS1 | 7.8 | 5.91 |
| Wilson Park Creek near 13^th^ Street | 04087148 | DS2 | 19.7 | 3.65 |
| Wilson Park Creek upstream from St. Luke’s Hospital | (040871488) | DS3 | 30.6 | 0.56 |
| Wilson Park Creek at St. Luke’s Hospital gage | 040871488 | DS3-gage | 30.8 | 0 |

^a^ National Water Information System
