# Supplementary material for: Advanced biofilm analysis in streams receiving organic deicer runoff
Source: PLoS One. 2020 Jan 22;15(1):e0227567. doi: 10.1371/journal.pone.0227567 (PMC6975536; doi:10.1371/journal.pone.0227567)
Supplement: S2 Table — (DOC) [file pone.0227567.s003.doc]

**S2 Table. Physical, water-quality,** and biofilm parameters used in stepwise linear regression modeling.

| **Dataset** | **Description** | **Units** | **Instantaneous** | **Summary statistics, 0.5–20 weeks^a^** | **Flow-weighted mean concentration, 2–20 weeks^b^** |
| --- | --- | --- | --- | --- | --- |
| Site indicator term | DS1**^c^**, binary sample indicator field | n/a |  |  |  |
|  | DS2**^c^**, binary sample indicator field | n/a |  |  |  |
| Physical measurement | Ice cover, percent of reach | percent | x |  |  |
|  | Ice cover, average reach thickness | centimeter | x |  |  |
| Water quality | pH | standard units | x |  |  |
|  | Specific conductance (SC) | microsiemens per cubic centimeter at 25 degrees Celsius | x^d^ |  |  |
|  | Temperature, water | degrees Celsius | x | x |  |
|  | Chemical oxygen demand (COD) concentration | milligrams per liter | x^d^ |  | x^d^ |
|  | Total Kjeldahl nitrogen (TKN) | milligrams per liter as nitrogen | x^d^ |  |  |
|  | Nitrate + nitrite | milligrams per liter as nitrogen | x^d^ |  |  |
|  | Total phosphorus | milligrams per liter as phosphorus | x^d^ |  |  |
|  |  |  |  |  |  |
| ^a^ Mean, maximum, minimum, median, and standard deviation calculated over periods of 0.5, 1, 2, 4, 6, 8, 12, 16, and 20 weeks. | | | | | |
| ^b^ Calculated over periods of 2, 4, 6, 8, 12, 16 and 20 weeks. | | |  |  |  |
| ^c^ Site information is provided in S1 Table. | | |  |  |  |
| ^d^ Data were log (base 10) transformed. | |  |  |  |  |
|  | | |  |  |  |
