# Supplementary material for: Advanced biofilm analysis in streams receiving organic deicer runoff
Source: PLoS One. 2020 Jan 22;15(1):e0227567. doi: 10.1371/journal.pone.0227567 (PMC6975536; doi:10.1371/journal.pone.0227567)
Supplement: S3 Table — An X indicates that the analytical technique was performed on the sample. (DOC) [file pone.0227567.s004.doc]

**S3 Table. Inventory of analyses performed on biofilm samples.** An X indicates that the analytical technique was performed on the sample.

| **Site Identifier[[1]](#footnote-2)** | **Subsite** | **Sample Name** | **Sample Type** | **Collection Date** | **Time** | **Microscopy and qPCR** | **Microarray** | **Whole metagenome sequencing** | **Cultured Isolate** |
| --- | --- | --- | --- | --- | --- | --- | --- | --- | --- |
| **DS1** |  | DS1__20091223_1145 | Regular | 12/23/09 | 11:45 | X |  |  |  |
| **DS1** |  | DS1__20100128_1125 | Regular | 1/28/10 | 11:25 | X | X |  |  |
| **DS1** |  | DS1__20100224_1120 | Regular | 2/24/10 | 11:20 | X | X | X |  |
| **DS1** |  | DS1__20100318_1130 | Regular | 3/18/10 | 11:30 | X | X | X |  |
| **DS1** |  | DS1__20100330_1030 | Regular | 3/30/10 | 10:30 | X |  |  |  |
| **DS1** |  | DS1__20100330_1031 | Replicate | 3/30/10 | 10:31 | X | X |  |  |
| **DS1-gage** |  | DS1-gage__20100330_1120 | Regular | 3/30/10 | 11:20 | X | X |  |  |
| **US1** |  | US1__20100330_1445 | Regular | 3/30/10 | 14:45 | X | X |  |  |
| **DS2** | sandy mid-channel | DS2_sand_20100331_1445 | Regular | 3/31/10 | 14:45 | X | X |  |  |
| **DS2** | riprapped stream edge | DS2_riprap_20100331_1450 | Regular | 3/31/10 | 14:50 | X | X |  |  |
| **DS3** |  | DS3__20100331_1005 | Regular | 3/31/10 | 10:05 | X | X |  |  |
| **DS1** |  | DS1__20100412_1050 | Regular | 4/12/10 | 10:50 | X |  |  |  |
| **DS1** |  | DS1__20100427_1045 | Regular | 4/27/10 | 10:45 | X |  |  |  |
| **DS1** |  | DS1__20100525_1100 | Regular | 5/25/10 | 11:00 | X | X |  |  |
| **DS3** |  | DS3__20100617_1435 | Regular | 6/17/10 | 14:35 | X |  |  |  |
| **DS3** |  | DS3__20100617_1436 | Replicate | 6/17/10 | 14:36 | X |  |  |  |
| **DS2** | sandy mid-channel | DS2_sand_20100617_1200 | Regular | 6/17/10 | 12:00 | X |  |  |  |
| **DS2** | riprapped stream edge | DS2_riprap_20100617_1150 | Regular | 6/17/10 | 11:50 | X |  |  |  |
| **US1** |  | US1__20100618_1220 | Regular | 6/18/10 | 12:20 | X |  |  |  |
| **DS1** |  | DS1__20100618_1450 | Regular | 6/18/10 | 14:50 | X |  |  |  |
| **DS1-gage** |  | DS1-gage__20100618_1620 | Regular | 6/18/10 | 16:20 | X |  |  |  |
| **DS1** |  | DS1__20101103_1050 | Regular | 11/3/10 | 10:50 | X | X |  |  |
| **DS1** |  | DS1__20101215_1145 | Regular | 12/15/10 | 11:45 | X |  |  |  |
| **DS1** |  | DS1__20110112_1130 | Regular | 1/12/11 | 11:30 | X |  |  |  |
| **DS1** |  | DS1__20110214_1150 | Regular | 2/14/11 | 11:50 | X |  |  |  |
| **DS1** |  | DS1__20110303_1145 | Regular | 3/3/11 | 11:45 | X |  |  |  |
| **DS2** | sandy mid-channel | DS2_sand_20110330_1055 | Regular | 3/30/11 | 10:55 | X |  |  |  |
| **DS2** | riprapped stream edge | DS2_riprap_20110330_1050 | Regular | 3/30/11 | 10:50 | X |  |  |  |
| **US1** |  | US1__20110329_1500 | Regular | 3/29/11 | 15:00 | X |  |  |  |
| **US1** |  | US1__20110329_1501 | Replicate | 3/29/11 | 15:01 | X |  |  |  |
| **DS1-gage** |  | DS1-gage__20110329_1215 | Regular | 3/29/11 | 12:15 | X |  |  |  |
| **DS1** |  | DS1__20110329_1055 | Regular | 3/29/11 | 10:55 | X |  |  |  |
| **DS3** |  | DS3__20110330_1305 | Regular | 3/30/11 | 13:05 | X |  |  |  |
| **DS1** |  | DS1__20110503_1045 | Regular | 5/3/11 | 10:45 | X |  |  |  |
| **DS2** | sandy mid-channel | DS2_sand_20110601_1135 | Regular | 6/1/11 | 11:35 | X |  |  |  |
| **DS2** | sandy mid-channel | DS2_sand_20110601_1136 | Replicate | 6/1/11 | 11:36 | X |  |  |  |
| **DS2** | riprapped stream edge | DS2_riprap_20110601_1130 | Regular | 6/1/11 | 11:30 | X |  |  |  |
| **DS3** |  | DS3__20110601_0915 | Regular | 6/1/11 | 9:15 | X |  |  |  |
| **US1** |  | US1__20110601_1730 | Regular | 6/1/11 | 17:30 | X |  |  |  |
| **DS1** |  | DS1__20110601_1430 | Regular | 6/1/11 | 14:30 | X |  |  |  |
| **DS1-gage** |  | DS1-gage__20110601_1515 | Regular | 6/1/11 | 15:15 | X |  |  |  |
| **DS1** |  | DS1__20110629_1345 | Regular | 6/29/11 | 13:45 | X |  |  |  |
| **DS1** | (296 meters downstream from DS1 site) | DS1__20140531_1745 | Regular | 5/31/14 | 17:45 |  |  |  | X |

1. Site information is provided in S1 Table. [↑](#footnote-ref-2)
