# Supplementary material for: Advanced biofilm analysis in streams receiving organic deicer runoff
Source: PLoS One. 2020 Jan 22;15(1):e0227567. doi: 10.1371/journal.pone.0227567 (PMC6975536; doi:10.1371/journal.pone.0227567)
Supplement: S4 Table — (DOC) [file pone.0227567.s005.doc]

**S4 Table. Predictors selected in multiple linear regressions for explaining variability in heterotroph biofilm prevalence and dissolved oxygen (DO)** concentrations.

| Response variable | Predictor variables (sign of coefficient) |
| --- | --- |
|  | Mean 2-week flow-weighted COD concentration ^a^ (+) |
| Heterotrophic biofilm volume ^a^ | DS1^b^, binary sample indicator field (+) |
|  | DS2^b^, binary sample indicator field (+) |
|  | Heterotrophic biofilm volume ^a^ (-) |
| DO concentration | Maximum 2-week water temperature (-) |
|  | Mean 8-week flow-weighted COD concentration ^a^ (-) |

^a^ Parameter was log-10 transformed in the regression equation.

^b^ Site information is provided in S1 Table.
