# Supplementary material for: Advanced biofilm analysis in streams receiving organic deicer runoff
Source: PLoS One. 2020 Jan 22;15(1):e0227567. doi: 10.1371/journal.pone.0227567 (PMC6975536; doi:10.1371/journal.pone.0227567)
Supplement: S5 Table — (DOC) [file pone.0227567.s006.doc]

**S5 Table. Strongest Spearman rank correlations observed for ratios of *sthA* DNA to 16S rDNA.**

| Variable 1 | Variable 2 | rho^a^ | p^b^ | n^c^ |
| --- | --- | --- | --- | --- |
|  | Heterotrophic biofilm volume | +0.73 | 1.5E-7 | 38 |
| *sthA* DNA:16S rDNA | Total phosphorus | +0.56 | 2.7E-4 | 37 |
|  | 2-week, flow-weighted chemical oxygen demand (COD) | +0.53 | 1.4E-3 | 34 |
|  | Grab sample COD | +0.52 | 9.1E-4 | 37 |
|  | 8-week, flow-weighted COD | +0.52 | 1.7E-3 | 34 |
|  | 4-week, flow-weighted COD | +0.48 | 4.4E-3 | 34 |
|  | 6-week, flow-weighted COD | +0.47 | 5.4E-3 | 34 |
|  | 12-week, flow-weighted COD | +0.45 | 7.5E-3 | 34 |
|  | Dissolved oxygen (DO) concentration | -0.77 | 1.4E-8 | 38 |
|  | Aggregated autotrophic biofilm volume | -0.55 | 3.4E-4 | 38 |
|  | Dissolved nitrate+nitrite | -0.51 | 1.2E-3 | 37 |

^a^ Spearman rank correlation coefficient indicating the direction and strength of variable association.

^b^ Probability that the null hypothesis is true.

^c^ Number of samples considered in the correlation.
