# Supplementary figures and images for: Advanced biofilm analysis in streams receiving organic deicer runoff
Source: PLoS One. 2020 Jan 22;15(1):e0227567. doi: 10.1371/journal.pone.0227567 (PMC6975536; doi:10.1371/journal.pone.0227567)

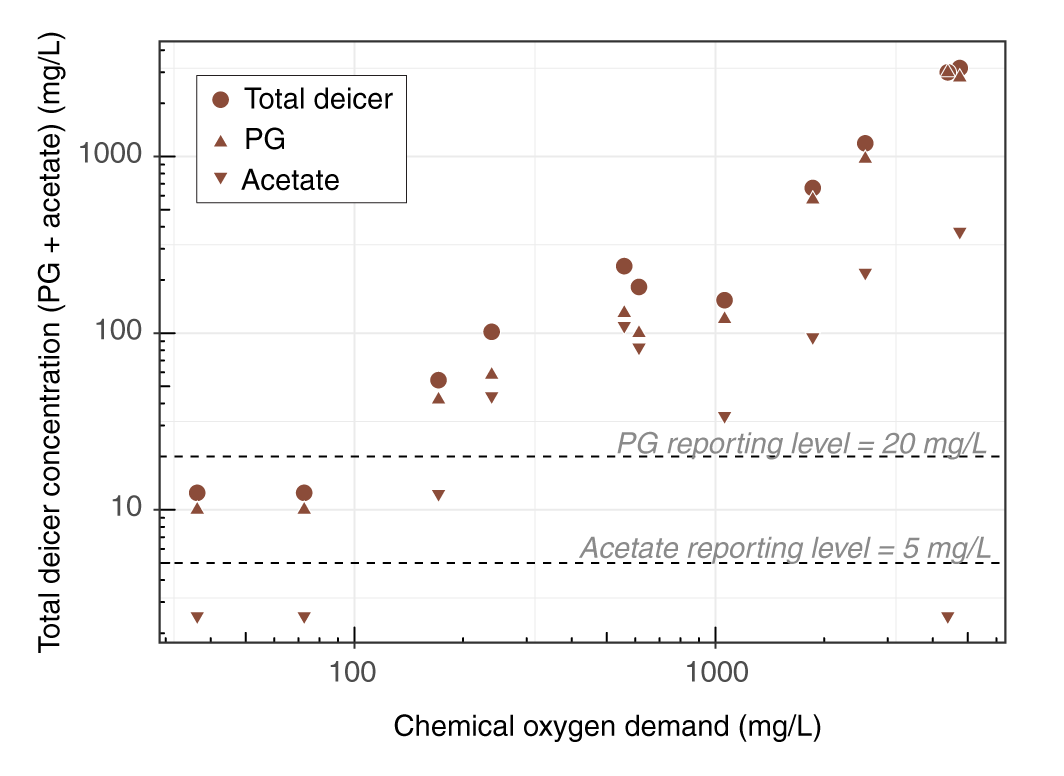

Supplement: S1 Fig — All samples shown here are flow-composite samples. For the purposes of this graph, left-censored deicer concentrations are displayed at one-half the reporting level (5 mg/L for acetate, and 20 mg/L for PG). (TIF) [file pone.0227567.s007.tif]

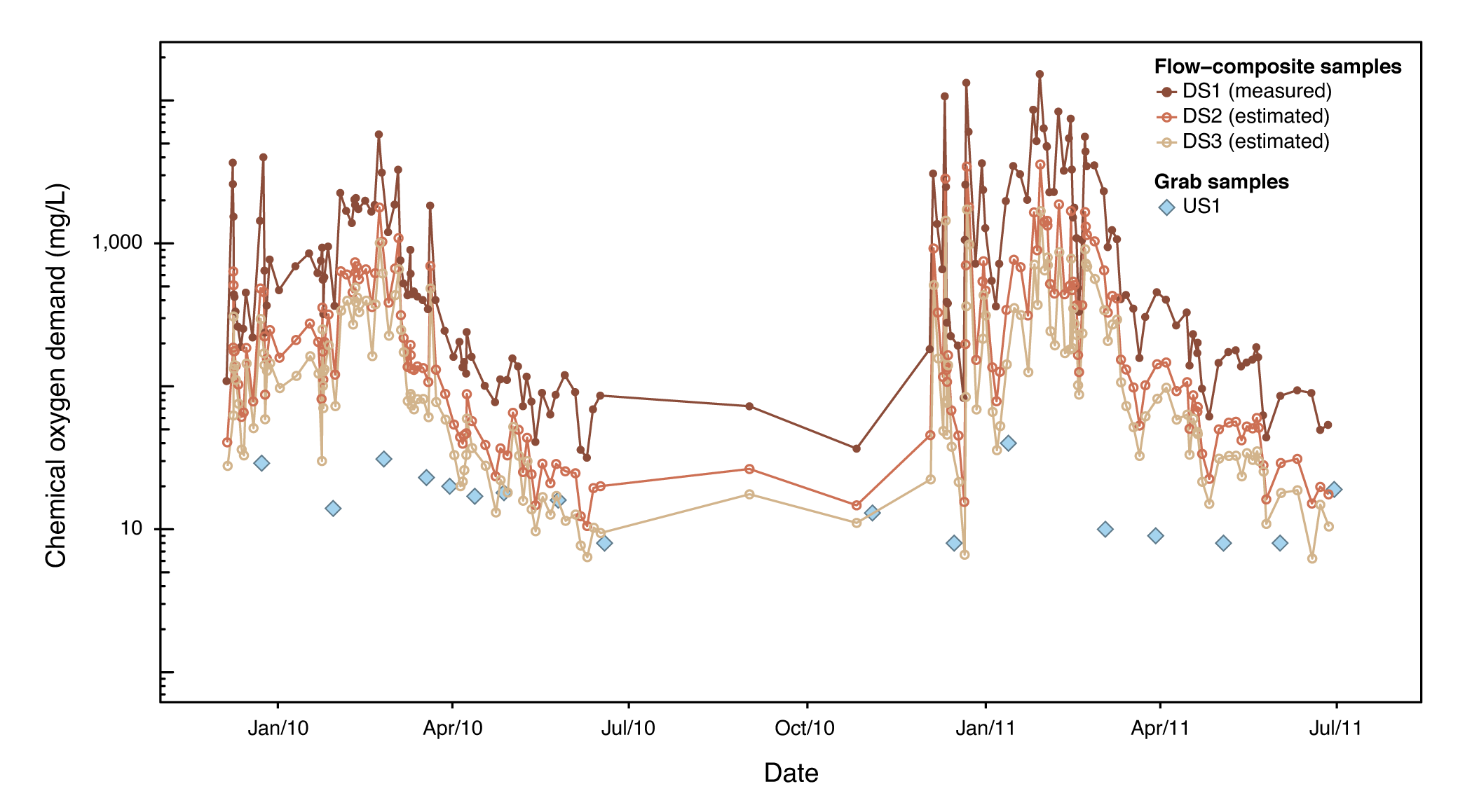

Supplement: S2 Fig — Values were measured at DS1 and estimated at DS2 and DS3. Concentrations for grab samples collected upstream from the airport, at US1, are also included for comparison. (TIF) [file pone.0227567.s008.tif]

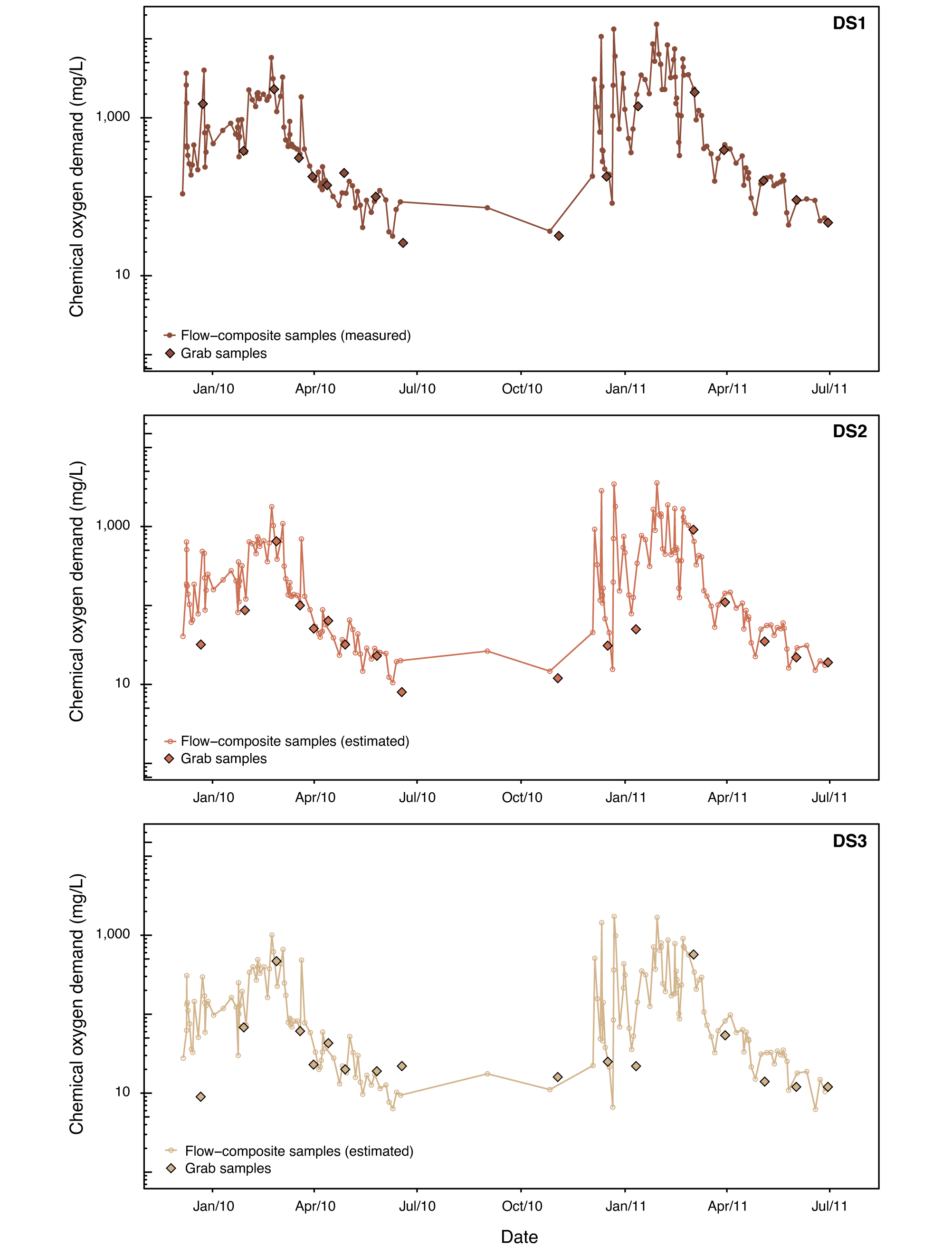

Supplement: S3 Fig — Flow-composite sample concentrations were measured at DS1 and estimated at DS2 and DS3. (TIF) [file pone.0227567.s009.tif]

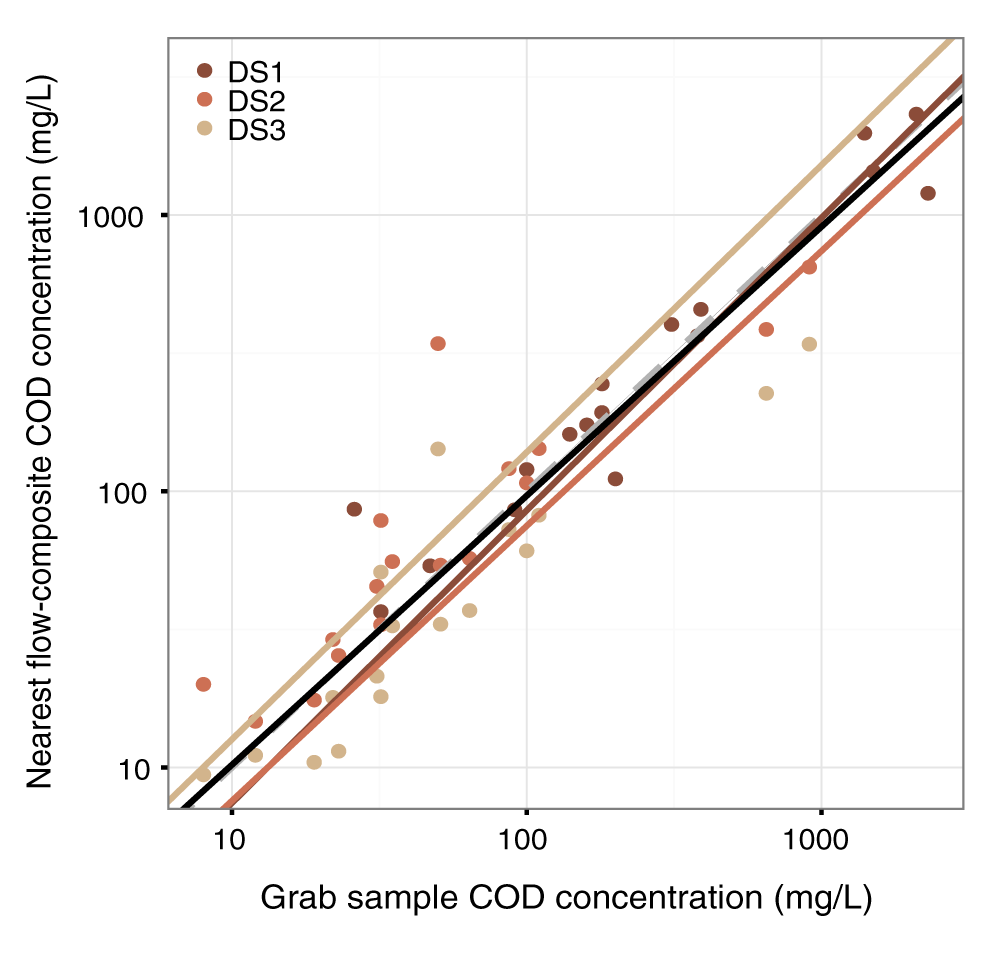

Supplement: S4 Fig — Dashed gray line, 1:1 relation; solid black line, regression (in log10; R2 = 0.86) across all sites and samples. Dark brown points and line, data and regression (in log10; R2 = 0.92) between samples collected at DS1; medium brown points and line, data and regression (in log10; R2 = 0.80) between samples collected at DS2; light brown points and line, data and regression (in log10; R2 = 0.83) between samples collected at DS3. (TIF) [file pone.0227567.s010.tif]
