# Supplementary material for: Advanced biofilm analysis in streams receiving organic deicer runoff
Source: PLoS One. 2020 Jan 22;15(1):e0227567. doi: 10.1371/journal.pone.0227567 (PMC6975536; doi:10.1371/journal.pone.0227567)
Supplement: Attachment — Submitted filename: ResponseToReviewers.docx [file pone.0227567.s012.docx]

Date:	December 18, 2019

To:	Dr. Steven Loiselle, Academic Editor, PLOS ONE

From:	Michelle A. Nott, USGS Upper Midwest Water Science Center, Middleton, WI

Subject:	Submission of revised manuscript “Advanced biofilm analysis in streams receiving organic deicer runoff”

Specific point-by-point responses to PLOS ONE reviewer comments are detailed below in blue.

Sincerely,

Michelle A. Nott

# **PLOS ONE editor email and reviewers' comments:**

PONE-D-19-14510R1

Advanced biofilm analysis in streams receiving organic deicer runoff

PLOS ONE

Dear Ms. Nott,

Thank you for submitting your manuscript to PLOS ONE. After careful consideration, we feel that it has merit but does not fully meet PLOS ONE’s publication criteria as it currently stands. Therefore, we invite you to submit a revised version of the manuscript that addresses the minor points raised during the review process. These are limited to those suggested by Reviewer 2 regarding the primer design and a couple additional comments.

We would appreciate receiving your revised manuscript by Jan 11 2020 11:59PM. When you are ready to submit your revision, log on to https://www.editorialmanager.com/pone/ and select the 'Submissions Needing Revision' folder to locate your manuscript file.

If you would like to make changes to your financial disclosure, please include your updated statement in your cover letter.

To enhance the reproducibility of your results, we recommend that if applicable you deposit your laboratory protocols in protocols.io, where a protocol can be assigned its own identifier (DOI) such that it can be cited independently in the future. For instructions see: http://journals.plos.org/plosone/s/submission-guidelines#loc-laboratory-protocols

Please include the following items when submitting your revised manuscript:

A rebuttal letter that responds to each point raised by the academic editor and reviewer(s). This letter should be uploaded as separate file and labeled 'Response to Reviewers'.

A marked-up copy of your manuscript that highlights changes made to the original version. This file should be uploaded as separate file and labeled 'Revised Manuscript with Track Changes'.

An unmarked version of your revised paper without tracked changes. This file should be uploaded as separate file and labeled 'Manuscript'.

Please note while forming your response, if your article is accepted, you may have the opportunity to make the peer review history publicly available. The record will include editor decision letters (with reviews) and your responses to reviewer comments. If eligible, we will contact you to opt in or out.

We look forward to receiving your revised manuscript.

Kind regards,

Steven Arthur Loiselle

Academic Editor

PLOS ONE

[Note: HTML markup is below. Please do not edit.]

Reviewers' comments:

Reviewer's Responses to Questions

Comments to the Author

1. If the authors have adequately addressed your comments raised in a previous round of review and you feel that this manuscript is now acceptable for publication, you may indicate that here to bypass the “Comments to the Author” section, enter your conflict of interest statement in the “Confidential to Editor” section, and submit your "Accept" recommendation.

Reviewer #1: All comments have been addressed

Reviewer #2: (No Response)

2. Is the manuscript technically sound, and do the data support the conclusions?

The manuscript must describe a technically sound piece of scientific research with data that supports the conclusions. Experiments must have been conducted rigorously, with appropriate controls, replication, and sample sizes. The conclusions must be drawn appropriately based on the data presented.

Reviewer #1: Yes

Reviewer #2: Yes

3. Has the statistical analysis been performed appropriately and rigorously?

Reviewer #1: Yes

Reviewer #2: Yes

4. Have the authors made all data underlying the findings in their manuscript fully available?

The PLOS Data policy requires authors to make all data underlying the findings described in their manuscript fully available without restriction, with rare exception (please refer to the Data Availability Statement in the manuscript PDF file). The data should be provided as part of the manuscript or its supporting information, or deposited to a public repository. For example, in addition to summary statistics, the data points behind means, medians and variance measures should be available. If there are restrictions on publicly sharing data—e.g. participant privacy or use of data from a third party—those must be specified.

Reviewer #1: Yes

Reviewer #2: Yes

5. Is the manuscript presented in an intelligible fashion and written in standard English?

PLOS ONE does not copyedit accepted manuscripts, so the language in submitted articles must be clear, correct, and unambiguous. Any typographical or grammatical errors should be corrected at revision, so please note any specific errors here.

Reviewer #1: Yes

Reviewer #2: Yes

6. Review Comments to the Author

Please use the space provided to explain your answers to the questions above. You may also include additional comments for the author, including concerns about dual publication, research ethics, or publication ethics. (Please upload your review as an attachment if it exceeds 20,000 characters)

Reviewer #1: (No Response)

Reviewer #2: I served as reviewer 2 on the prior submission of this manuscript. In my opinion the authors have effectively addressed the issues raised by myself and reviewer 1. The current version of the manuscript is significantly improved over the prior version. In particular, the authors' explanations of the use of the Phylo Chip and the metagenomic sequencing data are very helpful.

My only specific criticism is that the section on primer design (lines 323 to 28) does not provide enough detail, e.g. software used, the specific primer sequences, the size of the amplicon, etc. Thank you for bringing this to our attention. Additional detail describing primer design has been included in both the main text and appendix.

I have a few other minor comments that the authors could address:

Line 32 Change "with one previously identified" to "with a previously identified sthA sequence" I agree, this is much clearer; this edit has been made.

Line 33 Replace "RTqPCR" with "quantitative PCR". The text has been updated to read ‘quantitative real-time PCR.’ The addition of ‘real-time’ to this suggestion was an important distinction for one of the coauthors and doesn’t change the meaning here.

Overall, I understand that this was likely just a request to avoid using potentially confusing acronyms in the abstract, however, it made me wonder about our use of RTqPCR as an acronym for quantitative real-time PCR. The change from qPCR to RTqPCR was made during our last round of edits at the behest of a coauthor (i.e., not one of the reviewers). However, in looking at the literature it appears that we were incorrect in making this change, and that the scientific agreement for this acronym (i.e., RTqPCR) is to use it for referring to reverse transcription qPCR [1]. As such, all instances of RTqPCR in this manuscript (and the supporting Data Release) have been changed to qPCR.

Line 35 The phrase "stimulated by antecedent chemical oxygen demand concentrations" does not specify a positive or negative relationship. I would change this phrase either to "stimulated by elevated antecedent chemical oxygen demand concentrations" or "was positively correlated with antecedent chemical oxygen demand concentrations". Good catch; thank you for the suggested wording. I’ve edited text to match your first suggestion here.

Line 39 Remove the word "characteristics". This edit has been made.

Line 45 Remove the word "characteristics". This edit has been made.

Line 106 Remove the word "on". Good catch; this edit has been made.

Line 262 Change "was" to "were". Good catch; this edit has been made.

Line 265 The phrase "described lab temperatures" is unclear. Perhaps "temperatures described above" would be more clear. I agree, this is much clearer; this edit has been made.

Line 337 Replace "RTqPCR" with "Quantitative PCR". The text has been updated to read ‘Quantitative real-time PCR.’ I have also updated the RTqPCR header in the Results section to read the same (line 541).

Line 338 Change "estimate" to "quantify". This edit has been made.

7. PLOS authors have the option to publish the peer review history of their article (what does this mean?). If published, this will include your full peer review and any attached files.

If you choose “no”, your identity will remain anonymous but your review may still be made public.

Do you want your identity to be public for this peer review? For information about this choice, including consent withdrawal, please see our Privacy Policy.

Reviewer #1: No

Reviewer #2: No

[NOTE: If reviewer comments were submitted as an attachment file, they will be attached to this email and accessible via the submission site. Please log into your account, locate the manuscript record, and check for the action link "View Attachments". If this link does not appear, there are no attachment files to be viewed.]

While revising your submission, please upload your figure files to the Preflight Analysis and Conversion Engine (PACE) digital diagnostic tool, https://pacev2.apexcovantage.com/. PACE helps ensure that figures meet PLOS requirements. To use PACE, you must first register as a user. Registration is free. Then, login and navigate to the UPLOAD tab, where you will find detailed instructions on how to use the tool. If you encounter any issues or have any questions when using PACE, please email us at figures@plos.org. Please note that Supporting Information files do not need this step.

In compliance with data protection regulations, you may request that we remove your personal registration details at any time. (Remove my information/details). Please contact the publication office if you have any questions.

# **References cited:**

1.	Bustin SA, Benes V, Garson JA, Hellemans J, Huggett J, Kubista M, et al. The MIQE Guidelines: Minimum Information for Publication of Quantitative Real-Time PCR Experiments. Clinical Chemistry. 2009;55: 611–622. doi:10.1373/clinchem.2008.112797
